# Temporal artery biopsy: time for a rethink on training?

**DOI:** 10.1038/s41433-022-01963-1

**Published:** 2022-02-21

**Authors:** Georgia Osei, Paul Rainsbury, Daniel Morris, Anjana Haridas

**Affiliations:** 1 School of Medicine, Cardiff University, Heath Park, Cardiff, CF14 4XN UK; 2 Department of Ophthalmology, University Hospitals Plymouth NHS Trust, Plymouth, PL6 8DH UK; 3 Department of Ophthalmology, Cardiff and Vale University Health Board, Cardiff, CF14 4XW UK

**Keywords:** Education, Anatomy

## Abstract

**Background:**

Temporal artery biopsy (TAB) is often performed by ophthalmology trainees without direct supervision. The traditional model of ‘see one, do one, teach one’ still prevails in most units. Whilst it is generally a safe procedure, damage to the temporal branch of the facial nerve has been reported when harvesting the frontal branch of the superficial temporal artery.

**Methods:**

A survey of trainees from Wessex, Wales, London and Severn deaneries was performed to look at current training techniques, anatomical knowledge and practice.

**Results:**

38 trainees responded to the survey, with complete responses from 28 participants. Formal teaching of the anatomical considerations in TAB was not reported by any trainee, with informal teaching being standard practice. Whilst 61% of respondents reported having learnt about the anatomical ‘danger zone’ for facial nerve damage, 97% of trainees chose an incision that fell within this zone when given a choice between potential incision sites.

**Conclusion:**

TAB remains a largely trainee-taught, trainee-performed procedure. Most trainees are not aware of how to avoid the risk of damage to the temporal branch of the facial nerve. We suggest harvesting the parietal branch of the temporal artery via an incision outside the anatomical ‘danger zone’. In our experience, this is an easily taught technique that minimises the potential risk of damage to the frontal branch of the facial nerve.

## Introduction

Despite recent advances in investigative options for the diagnosis of giant cell arteritis (GCA), temporal artery biopsy (TAB) remains a vital diagnostic tool [1]. Efforts to reduce the ophthalmic complications of GCA, particularly blindness, have been addressed with guidelines that recommend urgent referral for TAB and prompt initiation of high dose steroids [2]. One study reported vision loss in 1 in 12 patients 6 months after diagnosis [3], highlighting the need for immediate diagnosis and the initiation of treatment. However, despite clear guidance on the requirement for TAB, most ophthalmology trainees rely on informal, often peer led, surgical training on the technique and lack formal teaching. Even then, a trainee may not attempt their first TAB until later in their training depending on the frequency of cases. Furthermore, there is often variation in who performs TAB in different units as vascular, general and ophthalmic surgeons frequently do so [4], thus diluting the case load for some trainees. Very few complications have been reported following temporal artery biopsy. However, the procedure is not without risk as damage to the temporal branch of the facial nerve has been reported in the literature [5–8]. One study of 75 TABs found ongoing frontalis weakness in 10% of patients at 6 months, reducing to 3% at a year [9]. This may result in brow droop, as a result of impaired function of the frontalis muscle, causing permanent disfigurement [9].

**Fig. 1:**
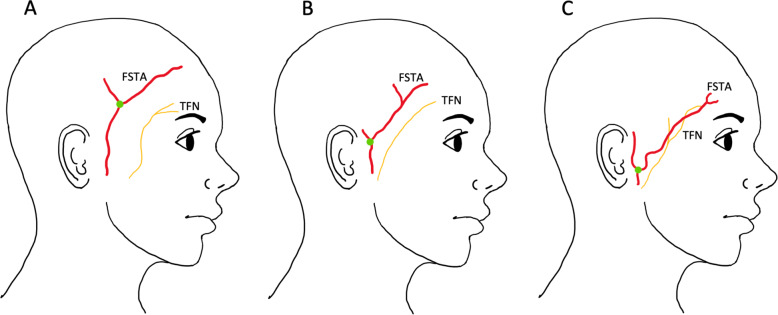
Illustration demonstrating anatomical variation in the relationship of the TFN and FSTA. **A** Most commonly found in over 72.7% of cadavers; **B** found in 20% of cadavers; **C** found in 7.3% of cadavers. The green dot denotes the bifurcation point of the superficial temporal artery. This figure is redrawn from, Surgical anatomy of the superficial temporal artery to prevent facial nerve injury during arterial biopsy (ref. [10]).

The superficial temporal artery (STA) lies within the superficial temporal fascia (STF) and the temporal branch of the facial nerve (TFN) courses deeper within the fascia’s fibrofatty layer [10]. The TFN is responsible for innervating the orbicularis oculi and frontalis muscle which are responsible for closing the eye and raising the eyebrow, respectively [4]. In some cases, the TFN can lie directly underneath the frontal branch of the superficial temporal artery (FSTA), putting it at increased risk of damage (Fig. 1) [7, 10]. Therefore, it is essential that an approach that avoids iatrogenic damage to the TFN is utilised.

**Fig. 2:**
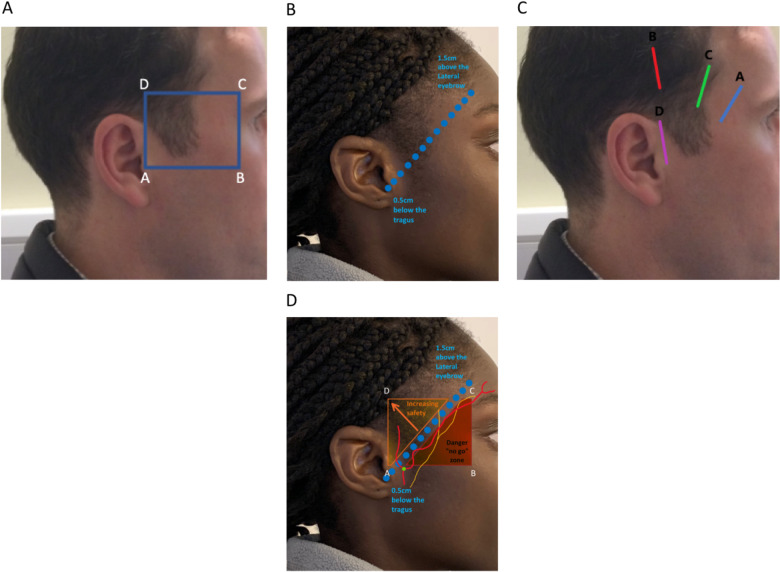
Surface markings of landmarks and surgical incision points related to TAB. **A** The anatomical ‘danger zone’. (A) the tragus of the ear, (B) the junction between the zygomatic arch and lateral orbital rim, (C) a point 2 cm above the superior orbital rim and (D) an area superior to the tragus that is in horizontal alignment with C. **B** Diagram of Pitanguy’s line (0.5 cm below the tragus to 1.5 cm above the lateral extremity of the eyebrow). A landmark that describes the superficial course of the temporal branch of the facial nerve. **C** Temporal artery biopsy incision options. **D** Diagram illustrating the anatomical ‘danger zone’, Pitanguy’s line and the course of the temporal artery and the facial nerve. The anatomical ‘danger zone’ can be divided into two triangles representing different levels of risk. The inferior red triangle represents the most at-risk area (‘no go zone’) and the orange triangle represents an area of increasing safety.

The anatomical ‘danger zone’ has been described as an area where the TFN and the FSTA are separated solely by the superficial temporal fascia [5]. Yoon et al. defined this area as (A) the tragus of the ear, (B) the junction between the zygomatic arch and lateral orbital rim, (C) a point 2 cm above the superior orbital rim and (D) an area superior to the tragus that is in horizontal alignment with C (Fig. 2A) [5]. Pitanguy’s line (Fig. 2B) is also a useful landmark and describes the superficial course of the TFN from 0.5 cm below the tragus to 1.5 cm above the lateral extremity of the eyebrow [11], however anatomical variants do exist. Avoidance of these areas is necessary to minimise the risk of damage to the TFN.

Other authors have previously described a technique for harvesting the parietal branch, rather than the frontal branch, of the temporal artery [5]. This can successfully be achieved by adapting the Gilles technique [12] and making a temporal incision 2.5 cm superior and anterior to the helix of the ear (Fig. 2C incision B) [13]. The sensitivity of harvesting the parietal branch of the temporal artery rather than the frontal branch has not been fully validated. However, recent interest in using ultrasound to diagnose GCA has demonstrated involvement of the parietal branch and it is now routinely included when scanning patients for suspected GCA suggesting that it is reasonable to use this branch [14].

In this study we investigated current training techniques, anatomical knowledge and practice in TAB to establish current practice.

## Methods

A survey was electronically distributed, using the Survey Monkey platform, to ~100 trainees from the Wessex, Wales, London and Severn deaneries during 2019.

Questions asked included year of training, number of TABs performed, and the method of training received. Trainees were also required to select where they would make their initial incision based on a series of diagrams and were asked about their awareness of the anatomical ‘danger zone’. All questions were designed to allow the respondents to select from options.

Our study adhered to the tenets of the Declaration of Helsinki. Consent was obtained from all individual participants included in the study.

## Results

38 responses were received out of approximately 100 distributed surveys. Ten respondents started the survey but only completed the first few questions and were therefore excluded from the analysis, leaving 28 complete responses. Responses were received from trainees from ST2 to ST7 level with most being from ST7 trainees.

The question concerning the type of training received revealed that 28 (100%) respondents received informal training from their colleagues in the format of ‘See one, do one, teach one’.

**Fig. 3:**
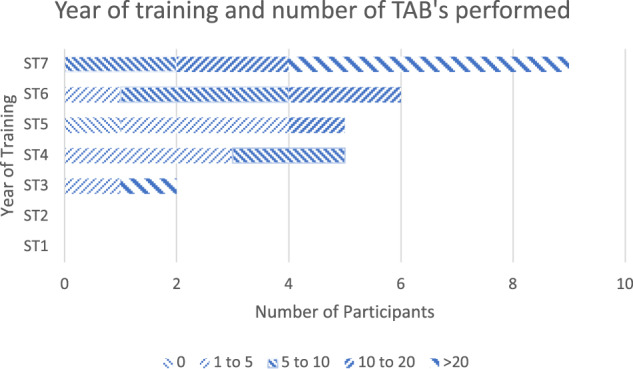
The number of temporal artery biopsies performed relative to the year of training. The majority of trainees had performed between 1 and 10 temporal artery biopsies. Surveys from ST2 trainees were amongst those excluded due to incomplete responses.

8/28 trainees (28.6%) had performed between 1 and 5 temporal artery biopsies; 8/28 (28.6%) had performed between 5 and 10 temporal artery biopsies; 5/28 (17.9%) had performed between 10 and 20 temporal artery biopsies; 6/28 (21.4%) had performed greater than 20 temporal artery biopsies, whilst 1/28 (3.6%) trainee had not yet performed a temporal artery biopsy as part of their training (Fig. 3).

Questions concerning key anatomical landmarks revealed that 17/28 (61%) reported having been taught about the concept of a ‘danger zone’ for injury to the temporal branch of the facial nerve (TFN) (Fig. 2A). 5/28 (17.9%) respondents were aware of Pitanguy’s line (Fig. 2B) whereas 27/28 (96.4%) respondents were able to correctly identify the ‘danger zone’ from the images provided. Interestingly however, when asked where they would make their initial incision, 27/28 (96.4%) respondents would choose to make their incision within the ‘danger zone’. (Table 1) shows possible incision points and the number of respondents that would select each incision (Fig. 2C).

**Table 1:** Possible incision options and the percentage of respondents that would choose each incision point. Incision C was the most common choice.

Incision	Response percent	Response count
A	7.1%	2
B	3.6%	1
C	89.3%	25
D	0%	0

## Discussion

This is the first multi-deanery study to investigate temporal artery biopsy training for ophthalmology trainees in the UK. Ophthalmologists in the UK are required to undertake a 7-year Ophthalmic specialist training programme which has an extensive curriculum comprising of a number of core learning outcomes that must be achieved by the end of the final year [15]. A key domain in this curriculum is surgical skills, and competency in temporal artery biopsy is a necessary requirement with the target year of achievement being year 7 [16]. Key outcomes of the skill include the consideration of risks and benefits of the procedure as well as a good understanding of landmarks and branches of the facial nerve [16].

At present, our results confirm that the most common approach to TAB training is in the format of ‘See one, do one, teach one’. This means that trainees are not receiving formal teaching. In other areas of ophthalmic surgical training such as cataract surgery, implementation of a structured surgical curriculum involving the use of wet labs and simulator training, has been demonstrated to reduce surgical complications [17]. Trainees might benefit from a structured course involving an e-learning tutorial with modules covering the contents and anatomical landmarks of the temporal region, the risk and benefits of the procedure and a step-by-step guide to the procedure. This may be followed by a video of the procedure and a supervised wet-lab experience before finally entering theatre. There are many online videos demonstrating different approaches to TAB [18, 19]. UK trainees may benefit from a teaching video approved by The Royal College of Ophthalmologists, and available on their website.

The use of informal teaching is likely multifactorial and may result from the shared performance of the procedure across multiple specialities. A recent retrospective cohort study reviewing the specialities performing TAB over a 10-year period in Canada found that general surgeons performed the most temporal artery biopsies which was closely followed by ophthalmologists and plastic surgeons [20]. It is not clear whether the UK experience follows this, but it is certainly the authors’ experience that in some units, ophthalmologists perform very few TABs. Lotfipour et al. evaluated trends in cataract surgery training curriculum and proposed that the choice of informal teaching may be a result of lack of faculty time and the perception that an apprentice-type approach to teaching negates the need for formal teaching [21]. However, in recent years there has been a shift away from an informal surgical apprenticeship model, largely as a result of restricted training hours and limitations in unsupervised experiential learning [22].

From the perspective of future practice, the use of informal teaching can lead to surgical procedures being influenced by the teaching surgeons’ personal preferences. This can result in less room to propose safer alternatives to a surgical technique. A safer approach to TAB would be to harvest the parietal branch of the superficial temporal artery, effectively eliminating the risk of damage to the facial nerve. This has previously been discussed by other authors [4], yet this technique is seldom used by ophthalmologists. The artery can be located and marked by following pulsations from the tragus, after removing a small amount of hair. A handheld doppler can also be used to confirm the course of the artery if required. Subsequently, TAB can be performed as normal.

While many trainees are performing temporal artery biopsies before their final year, with some performing greater than twenty biopsies, the majority of respondents indicated that they would make their initial incision within the ‘danger zone’. This would suggest that the teaching received by trainees lacks emphasis on the ‘danger zone’ as an anatomical region that should be avoided where possible. Therefore, we propose mapping the ‘danger zone’ during preoperative planning of TAB and avoiding the ‘danger zone’ when performing temporal artery biopsy.

Our survey reveals that the majority of trainees are aware of the anatomical ‘danger zone’, however less than one-fifth of respondents are aware of Pitanguy’s line. The concept of Pitanguy’s line does have its limitations, as the most at-risk temporal branch of the facial nerve typically has multiple rami crossing the zygomatic arch [23, 24]. However, marking it out preoperatively can help the surgeon to delineate a definite ‘no go’ zone (Fig. 2D) within the danger area that should be avoided, with increasing safety the more superior and lateral the incision point.

There are some limitations to this study. A primary limitation is the generalisation of these results. This is because the survey was distributed to a limited number of deaneries and in addition not all trainees responded. Furthermore, we received a number of incomplete surveys which were excluded from the analysis. Nonetheless, our study provides an insight into how trainees are being taught to perform a TAB and assists in evoking discussion regarding how training can be made safer.

## Conclusion

Despite the development of formal surgical teaching and simulation in other branches of ophthalmic surgery, the teaching of TAB continues to be taught to trainees, by trainees via the traditional apprenticeship method. Harm to patients may be avoided by raising awareness of the ‘danger zone’ and harvesting the parietal branch of the superficial temporal artery. Formal teaching on how to map the ‘danger zone’ and Pitanguy’s line and a demonstration of the above technique would be a beneficial addition to teaching trainees how to perform a TAB. This is particularly relevant as we are currently seeing an expansion in different methods of training, including the use of online resources, as a result of the COVID-19 pandemic. This has allowed us to be more creative and resourceful in the way we approach teaching which is proving to be beneficial in many sectors. TAB is a suitable technique that can be optimised with the addition of virtual training, and this will hopefully make the procedure safer for patients as well as instil confidence in our trainees.

### Summary

#### What was known before

- Temporal artery biopsy (TAB) is generally a safe procedure to perform however, there is a risk of damaging the temporal branch of the facial nerve. There is currently no formal teaching of the procedure to ophthalmology trainees. The traditional model of ‘see one, do one, teach one’ still prevails in most units.

#### What this study adds

- This study highlights that TAB still remains a primarily trainee-taught, trainee-performed procedure. Ophthalmology trainees will benefit from formal teaching on the anatomical danger zone and how best to avoid it.
